# Serum Leptin and Resistin Levels in Knee Osteoarthritis—Clinical and Radiologic Links: Towards Precise Definition of Metabolic Type Knee Osteoarthritis

**DOI:** 10.3390/biomedicines9081019

**Published:** 2021-08-15

**Authors:** Sevdalina Nikolova Lambova, Tsvetelina Batsalova, Dzhemal Moten, Stela Stoyanova, Elenka Georgieva, Lyudmila Belenska-Todorova, Desislava Kolchakova, Balik Dzhambazov

**Affiliations:** 1 Department of Propaedeutics of Internal Diseases, Faculty of Medicine, Medical University of Plovdiv, 4002 Plovdiv, Bulgaria; 2 Department of Rheumatology, MHAT “Sveti Mina”, 4000 Plovdiv, Bulgaria; 3 Department of Developmental Biology, Plovdiv University, Paisii Hilendarski, 4000 Plovdiv, Bulgaria; tsvetelina@uni-plovdiv.bg (T.B.); moten@uni-plovdiv.bg (D.M.); stela.stoyanova@uni-plovdiv.bg (S.S.); elenkageorgieva@uni-plovdiv.bg (E.G.); kolchakova@uni-plovdiv.bg (D.K.); balik@uni-plovdiv.bg (B.D.); 4 Faculty of Medicine, Sofia University, St. Kliment Ohridski, 1407 Sofia, Bulgaria; lbelenska@uni-sofia.bg

**Keywords:** knee osteoarthritis, adipokines, leptin, resistin, obesity, body mass index

## Abstract

Obesity is considered a major risk factor for the development and progression of knee osteoarthritis (OA). Apart from the mechanical effect of obesity via increase in mechanical overload of weight-bearing joints, an association with hand OA has been observed. There has been increasing interest in the role of adipokines in the pathogenesis of OA in the recent years. It has been suggested that their systemic effects link obesity and OA. In this regard, the aim of the current study was measurement and analysis of serum levels of leptin and resistin in patients with knee OA with different body mass index (BMI). Seventy-three patients with primary symptomatic knee OA at the age between 35 and 87 years (mean age 66 years) were included in the study (67 women and 6 men). The patients were from 2nd to 4th radiographic stage according to Kellgren–Lawrence scale. 43 patients were with concomitant obesity (BMI ≥ 30 kg/m^2^, mean values 38.34 ± 8.20) and 30 patients with BMI < 30 kg/m^2^ (mean values 25.07 ± 2.95). Eleven individuals with different BMIs, including cases with obesity but without radiographic knee OA, were examined as a control group. Serum levels of leptin and resistin were measured via ELISA method. In patients with knee OA and BMI ≥ 30 kg/m^2^, serum levels of leptin (39.546 ± 12.918 ng/mL) were significantly higher as compared with healthy individuals (15.832 ± 16.531 ng/mL, *p* < 0.05) and the patients with low BMI (*p* < 0.05). In patients with BMI < 30 kg/m^2^ the levels of leptin (13.010 ± 10.94 ng/mL) did not differ significantly from the respective values in the control group (*p* = 0.48). Serum levels of resistin were also higher in knee OA patients in comparison with healthy controls, but the difference was statistically significant only for patients with high BMI (2.452 ± 1.002 ng/mL in the group with BMI ≥ 30 kg/m^2^; 2.401 ± 1.441 ng/mL in patients with BMI < 30 kg/m^2^; 1.610 ± 1.001 ng/mL in the control group, *p* < 0.05). A correlation was found between the serum levels of leptin and radiographic stage of OA, i.e., higher leptin levels were present in the more advanced 3rd and 4th radiographic stage, while for resistin a correlation was observed in the patient subgroup with BMI < 30 kg/m^2^. Serum leptin and resistin levels and clinical characteristics were analyzed in patients with different clinical forms of OA. Novel clinical correlations have been found in the current study in patients with isolated knee OA vs. cases with presence of other disease localizations. It has been observed that patients with isolated knee OA were significantly younger and had higher BMI as compared with cases in whom OA is combined with other localizations i.e., spondyloarthritis ± presence of hip OA and with generalized OA. This supports the hypothesis that presence of obesity promotes earlier development of knee OA as an isolated localization of the disease in younger patients before appearance of osteoarthritic changes at other sites. The levels of leptin and resistin in isolated knee OA were also higher. Serum levels of leptin and resistin in combination with patients’ clinical characteristics suggest existence of different clinical and laboratory profile through which more precise definition of metabolic phenotype of knee OA would be possible. Considering the fact that obesity is a modifiable risk factor that has an impact on progression of knee OA, different approaches to influence obesity may offer potential for future disease-modifying therapeutic interventions.

## 1. Introduction

The knee is the most common localization of osteoarthritis (OA). Obesity is considered a major risk factor for its development and progression. Apart from the mechanical effect of obesity via increase in mechanical overload of weight-bearing joints, an association with hand OA has been observed [1,2,3]. The existence of different phenotypes of knee OA has been suggested. The high prevalence of different components of the metabolic syndrome in a proportion of patients with knee OA, i.e., obesity, diabetes, hypertension and dyslipidemia, as well as the association with some serum biomarkers, i.e., leptin, higher high-sensitivity CRP suggest the existence of a metabolic phenotype of knee OA [2]. There has been increasing interest in the role of adipokines in the pathogenesis of OA that are suggested to provide the link between obesity and OA. Adipokines represent mediators derived from dysfunctional adipose tissue, endothelial, immune cells, fibroblasts, and other cell types. Apart from their well-known role to regulate appetite, the feeling of satiety, fat distribution, insulin secretion, they also take a part in the regulation of endothelial function, inflammation, blood pressure, hemostasis. There is increasing evidence that adipokines modulate the immune system and are involved in the pathogenesis of OA and autoimmine diseases [4]. In in vitro studies, it has been observed that adipokines, such as leptin, adiponectin, visfatin and resistin, stimulate chondrolysis and inflammation [5,6].

Leptin is a peptide hormone that was initially described in 1994 and is mainly produced by adipose tissue [7]. Higher circulating leptin levels are found in obese individuals [7,8]. Currently, it is known that JAK/STAT signaling regulates leptin expression. It has been reported that elevated serum leptin levels could be found in OA and rheumatoid arthritis patients as compared with healthy subjects [7]. Leptin was also detected in synovial fluid of patients with rheumatoid arthritis [9] and OA and a positive correlation has been observed between its concentration in the synovial fluid and the severity of OA [7]. Higher leptin levels in osteoarthritic joint and expression of receptors for leptin on the surface of cartilage cells lead to the hypothesis that leptin may play a role in the cartilage degeneration in OA and may be the key molecule linking obesity to OA [7,10]. It has been shown that leptin has a catabolic effect on articular cartilage. Injection of recombinant leptin in the knee joints of rats lead to significant increase in both gene and protein levels of matrix metalloproteinases-2 (MMP), MMP-9, cathepsin D and decrease in basic fibroblast growth factor in the cartilage [11]. A correlation between leptin levels and prevalence of knee OA after adjustment for age, race/ethnicity and body mass index (BMI) has been demonstrated [12].

Resistin is a cysteine-rich polypeptide hormone with a molecular weight of 12 kDa, secreted by macrophages and adipocytes in humans and mice [13]. Resistin was first described in 2001 by Steppan et al. as a small circulating mouse protein that was expressed and secreted by adipocytes. Its serum levels were significantly increased in mouse models of genetic and diet-induced obesity and was found to be involved in the development of insulin resistance, thus mediating the link between obesity and diabetes [14,15]. In human blood, it circulates as a dimeric protein, consisting of two 92-amino acid polypeptides, and is predominantly expressed on macrophages [13]. It has been demonstrated that, in humans, a major source of resistin are cell types other than adipose cells, such as peripheral blood mononuclear cells, macrophages and bone marrow cells [16]. It has been shown that in humans resistin recruits immune cells and acts as a pro-inflammatory mediator. Higher resistin levels in both serum and joints, i.e., in the synovial fluid have been found in OA and in rheumatoid arthritis patients [17,18,19,20]. Predominant is the observation that resistin levels are higher in serum than in synovial fluid. It has been suggested that resistin inherits pro-inflammatory effects on human cartilage, promotes expression of enzymes involved in cartilage degradation and inhibition of matrix synthesis via several pathways, i.e., Toll-like receptor, 4 adenylyl cyclase-associated protein 1 receptor, nuclear factor-кB, etc. [13]. In a study by Wang et al. (2016) that included 194 patients with symptomatic knee OA, serum resistin level positively correlated with cartilage defects and bone marrow lesion on magnetic resonance imaging [21]. In a prospective multi-center study, plasma resistin levels positively correlated with radiographic early-stage knee OA and the association was independent of BMI. Knee radiographs were performed at baseline and after two and five years. Interestingly, plasma resistin level was associated with incidence of newly-diagnosed knee OA in individuals without OA at baseline. Of note, a correlation between plasma leptin level and radiographic progression of knee OA was observed, but the association was absent after adjustment for BMI [22]. In an in vitro study, it has been demonstrated that resistin increases the expression of vascular adhesion molecule-1 on human synovial fibroblasts in OA and facilitates the adhesion of monocytes to synovial fibroblasts. In a rat model of OA induced by anterior cruciate ligament transection, the inhibition of resistin activity prevented development of cartilage damage [23]. The effects of recombinant adipokines on subchondral bone tissue from femoral heads in hip OA patients obtained at the time of surgery of total hip arthroplasty was studied. After stimulation of subchondral bone from patients with normal body weight with recombinant leptin, resistin or visfatin, it has been observed that only resistin led to significant increase in abnormal type collagen that was also found in obese patients with hip OA [24].

The notion of a metabolic type of knee OA requires further precise definition of this desease phenotype based on a complex of clinical, laboratory and instrumental findings. The aim of the present study was measurement and analysis of serum levels of leptin and resistin in patients with knee OA with different BMIs and their links with clinical and radiological findings. 

## 2. Patients and Methods

### 2.1. Patients

Seventy-three patients with symptomatic primary knee OA at the age between 35 and 87 years (mean age 66 years) were included in the study (67 women and 6 men). Eleven individuals including patients with obesity but without radiographic knee OA (Kellgren-Lawrence = 0) were examined as a control group (Table 1). The control group was significantly younger than the patients with knee OA. This is related to the high frequency of radiographic knee OA in elderly, although asymptomaticindividuals. The patients with obesity were significantly younger in comparison with the subgroup with BMI < 30 kg/m^2^.

All patients underwent clinical examination and antero-posterior radiologic examination of knee joints bilaterally in a standing position. Inclusion criterion for participation in the study was presence of symptomatic and radiologically confirmed primary knee OA ≥ 2nd radiographic stage according to Kellgren–Lawrence scale [25]. Patients older than 35 years were included in the study due to evidence for increase in incidence of symptomatic knee OA in patients younger than 40 years [26]. Exclusion criteria included presence of different causes for secondary knee OA, i.e., posttraumatic knee OA, congenital or developmental diseases—either localized or generalized, inflammatory joint disease (rheumatoid arthritis, reactive arthritis, etc.), gout or other crystal arthropathies, history of septic arthritis, bone pathology or other diseases that could be associated with secondary knee OA [27]. All patients had bilateral knee involvement. The patients were from 2nd to 4th radiographic stage according to Kellgren–Lawrence scale. Patients, in whom the radiologic stage differed at the two knees, were classified into the more severe degree group. Presence or absence of OA with other localization was also documented. 

Generalized OA was defined as presence of multiple hand joints’ involvement, i.e., 3 or more hand joints (proximal interphalangeal joints—Bouchard’s nodes, distal interphalangeal joint—Heberden’s nodes, 1st carpo-metacarpal joint involvement) in association with large joint OA (knee and/or hip) and in the presence also of spondyloarthritis (cervical, thoracic or spine spondyloarthritis) [28]. 

According to the BMI values, the patients were divided into 2 groups, i.e., 43 patients were with concomitant obesity (BMI ≥ 30 kg/m^2^, mean values 38.68 ± 7.98) and 30 patients with BMI < 30 kg/m^2^ (mean values 25.03 ± 2.91). Pain was assessed using Western Ontario and McMaster Universities Arthritis Index (WOMAC) LK 3.1. The WOMAC pain scale consists of five items: (1) walking on flat ground; (2) going up or down stairs; (3) pain at night while in bed; (4) sitting or lying; and (5) standing upright. All components are scored on a scale of 0–4 i.e., none (0), mild (1), moderate (2), severe (3), and extreme (4). The scores for all items are summed up and the possible score ranges of 0 to 20 [29]. The study was approved by the local ethical committee and all the patients signed an informed consent. 

### 2.2. Assessment of Adipokines

Blood was drawn after 12 h fasting of the participants. Serum concentrations of leptin and resistin were measured uzing a human ELISA kit (BioVendor, Brno, Czech Republic) with a sensitivity of 30 pg/mL. All assays were performed according to the manufacturer’s instructions.

### 2.3. Statistical Analysis

Data were expressed as mean ± standard deviation (SD). Statistical analyses were performed by one-way ANOVA, unpaired *t*-test, Mann–Whitney U test, and Spearman rank correlation using StatView software (SAS Institute Inc., Cary, NC, USA). Differences were considered significant when *p*  <  0.05.

## 3. Results

### 3.1. Leptin and Resistin Levels in Knee OA Patients with Different BMI

**Figure 1:**
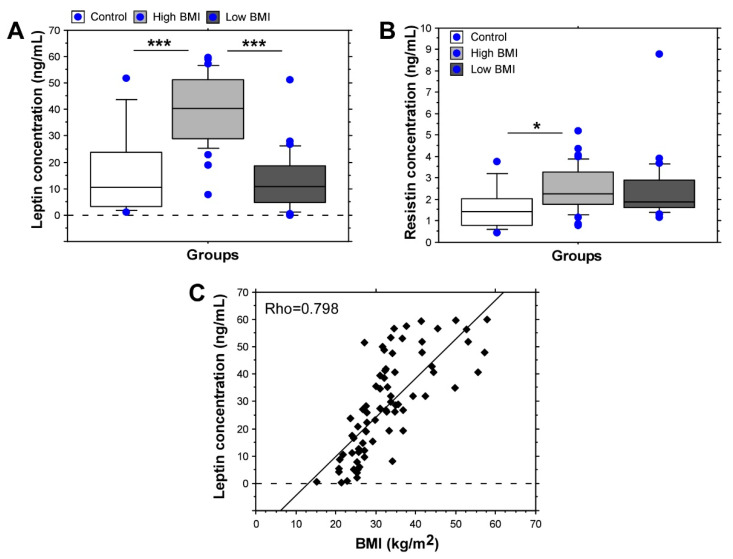
Leptin and resistin serum levels in knee OA patients. (**A**) Leptin concentration; (**B**) resistin concentration; (**C**) correlation between leptin serum levels and BMI; * *p* < 0,05; *** *p* < 0.001.

In patients with knee OA and BMI ≥ 30 kg/m^2^ serum levels of leptin (39.546 ± 12.918 ng/mL) were significantly higher as compared with the control group, which was comprised of individuals with different BMIs, including cases with obesity but without OA (15.832 ± 16.531 ng/mL, *p* < 0.05) (Figure 1A). Serum leptin levels in patients with high BMI were also significantly higher than those of patients with BMI < 30 kg/m^2^ (*p* < 0.05). In patients with BMI < 30 kg/m^2^, the levels of leptin (13.010 ± 10.94 ng/mL) did not differ significantly from the control group (*p* = 0.48). Serum levels of resistin were also higher in knee OA patients in comparison with controls and the difference reached statistical significance in patients with high BMI (2.452 ± 1.002 ng/mL in the group with BMI ≥ 30 kg/m^2^; 2.401 ± 1.441 ng/mL in patients with BMI < 30 kg/m^2^; 1.610 ± 1.001 ng/mL in the control group, *p* < 0.05) (Figure 1B). A strong correlation was observed between BMI and serum leptin level (Rho = 0.798), while for resistin an association was not found (Rho = 0.281) (Figure 1C).

### 3.2. Leptin and Resistin Levels and WOMAC-Pain Score in Knee OA

**Figure 2:**
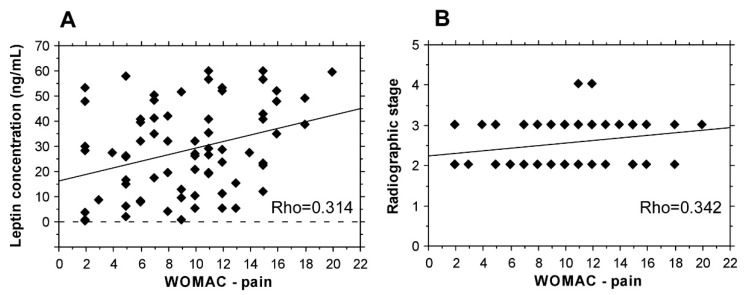
WOMAC-pain score correlations. (**A**) Association with leptin serum concentrations. (**B**) Correlation between WOMAC-pain score and radiographic stage.

A positive correlation between the levels of leptin and WOMAC-pain score was observed (Rho = 0.314) (Figure 2A), as well as between WOMAC-pain score and radiographic stage (Rho = 0.342) (Figure 2B). A significant association between resistin levels and WOMAC-pain score was not found (Rho = 0.121).

### 3.3. Association of Leptin and Resistin Levels with Radiographic Stage of Knee OA

**Figure 3:**
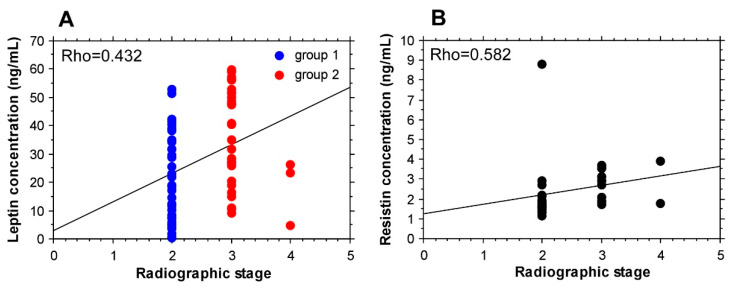
Radiographic stage correlations. (**A**) An association with leptin serum levels (group 1—2nd radiographic stage, group 2—3rd and 4th radiographic stage). (**B**) A correlation with resistin serum levels in patients with BMI < 30 kg/m^2^.

A correlation was found between the serum level of leptin and radiographic stage of OA, i.e., higher leptin levels were present in the more advanced 3rd and 4th radiographic stage of knee OA (Rho = 0.432), (Figure 3A), while for resistin there was not an association (Rho = 0.186). Interestingly, for resistin levels, a correlation with radiographic stage was observed only for the patients with BMI lower than 30 kg/m^2^ (Rho = 0.582), (Figure 3B). 

### 3.4. Clinical Associations in Knee OA with Different BMI

Interestingly, both serum levels of leptin and resistin were significantly higher as compared with the control group in patients with isolated knee OA and in cases combined with OA with other localization i.e., spondyloarthritis ± hip OA. There was no significant difference between cases with isolated knee OA and patients, in whom there was concomitant spondyloarthritis ± hip OA or with cases with generalized OA (Table 2). Comparing age in the group with high BMI ≥ 30 kg/m^2^ with different clinical forms of OA, it was found that patients with isolated knee OA were significantly younger (mean age 44.20 ± 6.68 years) vs. patients, in whom knee OA was combined with spondyloarthritis ± hip OA (mean age 64.41 ± 10.24 years, *p* < 0.05) and cases with generalized OA (mean age 71.25 ± 6.60 years, *p* < 0.05). BMI in patients with isolated knee OA was significantly higher as compared with the patients, in whom knee OA was combined with other localizations (Table 3).

**Figure 4:**
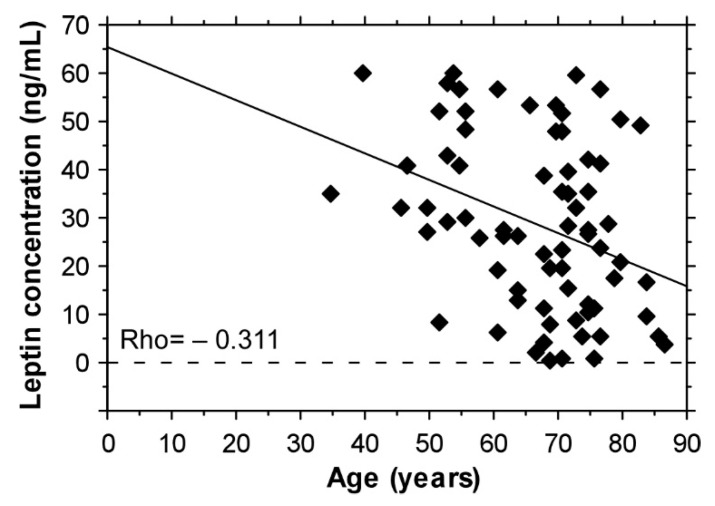
A tendency of decrease in serum leptin levels with aging.

**Figure 5:**
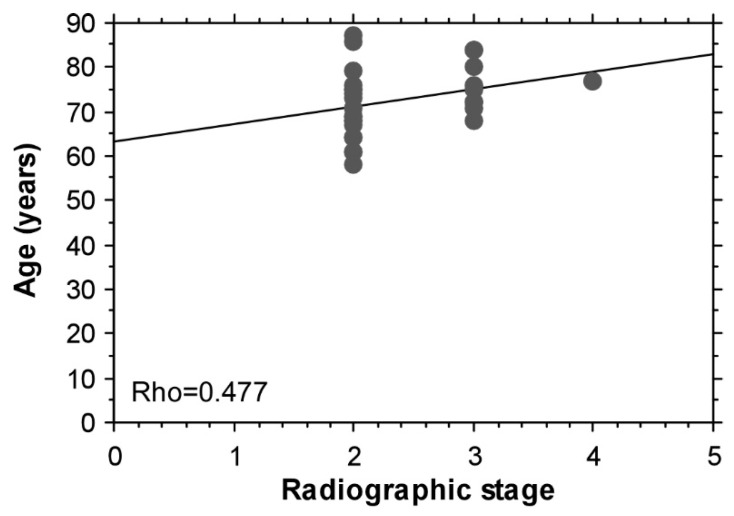
A correlation between age and radiographic stage of the knee OA in patients with BMI < 30 kg/m^2^.

A decrease of leptin with age was found (Rho = −0.311) (Figure 4). An association between age and the radiographic stage of OA was found only in the subgroup of patients with BMI < 30 kg/m^2^ (Rho = 0.477) (Figure 5).

## 4. Discussion

### 4.1. Leptin and Resistin Levels in Knee OA with Different BMI

Comparison of leptin levels in patients with knee OA with BMI < and ≥30 kg/m^2^ in the current study revealed significantly higher leptin levels only for obese patients with knee OA as compared with the control group. Of note, the control group included cases with different BMI together with patients with obesity but without radiographic knee OA. A strong correlation of leptin with BMI was observed. Resistin levels were higher in both groups in comparison with the control subjects but the difference was statistically significant only for the patient s with high BMI. In a large cross-sectional study with 6408 participants, comparison of leptin levels in isolated knee and isolated hand OA demonstrated that serum leptin levels were positively associated with OA and leptin mainly mediated the association of OA with obesity. The degree of mediation of association between high BMI and OA by leptin was higher in knee than in hand OA [30]. 

### 4.2. Leptin and Resistin Levels in Different Clinical Forms of OA

Adipokine levels in different clinical forms of knee OA have not been comparatively studied. In the current research, analysis of leptin and resistin serum levels in patients with different clinical forms of knee OA revealed presence of significant difference as compared with controls in both patients with isolated knee OA and in cases with concomitant presence of OA with other localization, i.e., spondyloarthritis ± presence of hip OA. Of note, there was no significant difference in adipokine levels between patients with isolated knee OA and patients, in whom there was concomitant spondyloarthritis ± hip OA or when compared with generalized OA. This finding indicates that elevated levels of leptin and resistin are mainly associated with joint involvement of the knee joint from osteoarthritic process and may be related to the large surface of synovial membrane of the knee joint or to be associated with other potential factors and requires further research. The results of the study suggest that elevated leptin and resistin levels in OA are associated mainly with the localization of the osteoarthritic process in the knee joint in the context of accompanying obesity, i.e., metabolic knee OA, which could be considered as a potential new component of the metabolic syndrome. 

Pathological changes in metabolic OA are suggested to be potentiated by the low-grade inflammation induced from the dysfunctional adipose tissue in obesity [31,32,33,34,35]. Of note, in an animal study with Wistar rats high-carbohydrate and high-fat diet led to obesity associated with spontaneous local inflammation of the synovial membranes that occurred before the cartilage degradation. Marked reduction in proteoglycan content was observed in articular cartilage [32]. 

### 4.3. Leptin and Resistin Levels and WOMAC-Pain Score in Knee OA

A positive correlation between the levels of leptin and WOMAC-pain score was observed as well as between radiographic stage and WOMAC-pain score. Similarly, Courties et al. found an association between leptin:adiponectin ratio and WOMAC-pain score in patients with knee and hip OA. Studying the association between level of different adipokines (leptin, adiponectin, visfatin, and resistin) and pain level in 150 patients with knee OA, a correlation was found for leptin and adiponectin [36]. In 115 patients with symptomatic primary knee OA, Calvet et al. also observed a positive association between leptin level and WOMAC-pain score as well as between leptin, resistin and osteopontin level and WOMAC-function score [37]. Thus, predominant is the observation about presence of correlation between leptin levels and pain intensity in OA. 

### 4.4. Leptin and Resistin Levels and Radiographic Stage of Knee OA

Interestingly, a correlation was found between the serum levels of leptin and radiographic stage of OA i.e., higher leptin levels were present in the more advanced 3rd and 4th radiographic stage of knee OA. Only for the patients with BMI lower than 30 kg/m^2^, an association between serum resistin levels and radiographic stage was present. Of note, in 74 patients with primary knee OA, Song et al. found a correlation between resistin measured in the synovial fluid and WOMAC-pain score, as well as with the radiographic stage of the disease, while for serum resistin levels (although higher than those in the synovial fluid) the association did not reach statistical significance that suggests also local release and effects of resistin [38]. Of note, an association between serum resistin levels and radiographic changes in hand OA has been also reported [39]. In a prospective study of Martel-Pelletier et al. (2016) in 138 patients with knee OA, adipokine levels were measured at baseline and after 24 months. Higher baseline values of leptin correlated with increased cartilage volume loss, while resistin level was not associated with cartilage volume loss i.e., with radiographic progression of knee OA [40]. In this regard, it could be concluded that both leptin and resistin promote disease progression and are associated with more severe morphological changes, i.e., with more advanced radiologic stage of OA. 

### 4.5. Clinical Associations in Knee OA with Different BMI

In the subgroup of patients with high BMI ≥ 30 kg/m^2^, cases with isolated knee OA were significantly younger as compared with patients, in whom knee OA was combined with spondyloarthritis ± presence of hip OA and with cases with generalized OA. Moreover, they were with significantly higher BMI when compared with other subgroups. These findings indicate that the presence of obesity and metabolic disturbances with the associated low-grade inflammation and increased adipokine levels lead to initial involvement of knee joints in the osteoarthritic process in younger patients. During evolution of the disease, many patients develop concomitant spondyloarthritis that is also age-related. While generalized OA predominates in elderly patients and is associated with other predominant pathogenic mechanisms, including specific genetic background [28]. 

The observed association between the age and the radiographic stage of knee OA only in the subgroup of patients with BMI < 30 kg/m^2^ suggests different pathogenic mechanisms in knee OA associated with normal BMI, which advances with aging, contrary to metabolic type of knee OA, which starts earlier in life.

In the current study, a decrease in leptin with the age was found. Similarly, in 470 healthy subjects (150 men and 320 women), Isidori et al. (2000) observed a gradual decline in leptin levels during aging and the reduction was greater in women than in men; it was independent of BMI and other age-related endocrine changes [41].

## 5. Conclusions

Serum levels of leptin and resistin could be used as potential biomarkers for identification of metabolic phenotype of knee OA. Their assessment in combination with other specific laboratory parameters, including markers for cartilage and bone turnover, could be used for evaluation of disease activity, further disease stratification, determination of disease progression rate and prognosis. Studies in this regard would provide the opportunity for precise definition of the profile of patients with metabolic type of knee OA and its differentiation from other subtypes of OA. Novel clinical correlations have been found in the current study in patients with isolated knee OA vs. cases with presence of other disease localizations. This supports the hypothesis that presence of obesity promotes earlier development of knee OA as an isolated localization of the disease in younger patients before appearance of osteoarthritic changes at other sites. Patients with isolated knee OA had significantly higher leptin and resistin levels as compared with the controls. Serum levels of leptin and resistin in combination with patients’ clinical characteristics suggest existence of different clinical and laboratory profile through which more precise definition of metabolic phenotype of knee OA would be possible. In addition, choosing a personalized therapeutic approach in different phenotypes of OA may offer potential for future disease-modifying therapeutic interventions. Considering the fact that obesity is a modifiable risk factor that has an impact on progression of knee OA, combination treatment with drugs that influence obesity, as well as cartilage and bone function may be a successful strategy in metabolic knee OA. 

## Figures and Tables

**Table 1:** Clinical and demographic characteristics of the patients with knee OA.

	Knee OA BMI ≥ 30 kg/m^2^ (Total n = 43)	Knee OA BMI ≤ 30 kg/m^2^ (Total n = 30)	Controls (n = 11)	
Age	62 ± 11 years 40 females, 3 males	72 ± 7 years 26 females, 4 males	72 ± 7 years 26 females, 4 males	Controls vs. patients with knee OA BMI ≥ 30 kg/m^2^ (*p* < 0.0001; Controls vs. patients with knee OA BMI < 30 kg/m^2^ (*p* < 0.0001); Patients with knee OA and BMI ≥ 30 kg/m^2^ vs. patients with knee OA and BMI < 30 kg/m^2^ (*p* = 0.0002)
BMI mean ± SD	38.68 ± 7.98 kg/m^2^	25.03 ± 2.91 kg/m^2^	27.87 ± 8.830 kg/m^2^	Controls vs. patients with knee OA BMI ≥ 30 kg/m^2^ (*p* < 0.0001; Controls vs. patients with knee OA BMI < 30 kg/m^2^ (*p* = 0.23); Patients with knee OA and BMI ≥ 30 kg/m^2^ vs. patients with knee OA and BMI < 30 kg/m^2^ (*p* < 0.0001)
Clinical forms	- n = 5 isolated knee OA - n = 4 generalized OA - n = 34 knee OA combined with spondyloarthritis and/or hip OA (including 26 patients with knee OA and spondyloarthritis and 8 patients with knee OA, spondyloarthritis and hip OA)	- n = 2 isolated knee OA - n = 5 generalized OA - n = 23 knee OA combined with spondyloarthritis		

**Table 2:** Leptin and resistin level in the presence of OA with different localization.

	Leptin	Resistin
- isolated knee OA n = 7 - generalized OA n = 9 - knee OA combined with spondyloarthritis and/or hip OA n = 57 - - controls n = 11	35.06 ± 20.11 ng/mL 23.43 ± 17.63 ng/mL 28.67 ± 17.66 ng/mL 15.83 ± 16.53 ng/ml	3.026 ± 0.776 ng/mL 1.908 ± 0.900 ng/mL 2.441 ± 1.250 ng/mL 1.610 ± 1.001 ng/ml
	*Controls vs. isolated knee OA p < 0.05;* *Controls vs. knee OA combined with spondyloarthritis ± hip OA p < 0.05;* Generalized OA vs. isolated knee OA *p* > 0.05: Knee OA combined with spondyloarthritis ± hip OA vs. isolated knee OA *p* > 0.05; Knee OA combined with spondyloarthritis ± hip OA vs. generalized OA *p* > 0.05	*Controls vs. isolated knee OA p < 0.05;* *Controls vs. knee OA combined with spondyloarthritis ± hip OA p < 0.05;* Generalized OA vs. isolated knee OA *p* > 0.05: Knee OA combined with spondyloarthritis ± OA vs. isolated knee OA *p* > 0.05; Knee OA combined with spondyloarthritis ± OA vs. generalized OA *p* > 0.05

**Table 3:** BMI in patients with different clinical forms of OA.

	BMI
- isolated knee OA (n = 7) - generalized OA (n = 9) - knee OA combined with spondyloarthritis ± hip OA (n = 57)	41.48 ± 13.68 kg/m^2^ 29.19 ± 3.45 kg/m^2^ 32.65 ± 8.83 kg/m^2^
*Generalized OA vs. isolated knee OA p < 0.05:* *Knee OA combined with spondyloarthritis* ± *hip OA vs. isolated knee OA p < 0.05;* Knee OA combined with spondyloarthritis ± hip OA vs. generalized OA *p* = 0.28	

## Data Availability

Data are contained within the article.
